# Secreted Phosphoprotein 1 in Lung Diseases

**DOI:** 10.3390/metabo15060365

**Published:** 2025-05-30

**Authors:** Hongli Liu, Cristian Coarfa, Arzoo N. Charania, Jennifer L. Larson-Casey, Ivan O. Rosas, Chao He

**Affiliations:** 1 Division of Pulmonary, Allergy, and Critical Care Medicine, Department of Medicine, University of Alabama at Birmingham, Birmingham, AL 35249, USA; hongliliu@uabmc.edu (H.L.); ancharan@uab.edu (A.N.C.); jennifercasey@uabmc.edu (J.L.L.-C.); 2 Department of Molecular and Cellular Biology, Baylor College of Medicine, Houston, TX 77030, USA; coarfa@bcm.edu; 3 Section of Pulmonary, Critical Care, and Sleep Medicine, Department of Medicine, Baylor College of Medicine, Houston, TX 77030, USA; ivan.rosas@bcm.edu

**Keywords:** SPP1, pulmonary fibrosis, interstitial lung diseases, lung cancer, COPD, asthma

## Abstract

Secreted phosphoprotein 1 (SPP1), also known as osteopontin (OPN) or early T lymphocyte activation protein 1 (ETA-1), is a multifunctional protein involved in numerous biological processes, including immune modulation, stress response, and tissue remodeling. The role of SPP1 in interstitial lung diseases (ILDs) has become an area of increasing interest, given its elevated expression in various ILDs such as idiopathic pulmonary fibrosis (IPF), connective tissue disease-associated ILD (CTD-ILD), and pneumoconiosis, especially with recent data derived from single-cell RNA sequencing. In addition to ILDs, SPP1 has been implicated in infectious granulomatous lung diseases, lung and pleural malignancies, airway diseases, and COVID-19. In most cases, higher SPP1 levels in serum, bronchoalveolar lavage fluid, or lung tissue carry a poor prognosis. SPP1 is expressed in multiple cells critical for fibrogenesis, including macrophages, epithelial cells, and fibroblasts, and SPP1 has emerged as a potential target for therapeutic interventions. Here, we review the proposed mechanisms by which SPP1 contributes to the development of lung disease, with an emphasis on ILD.

## 1. Introduction

**Figure 1:**
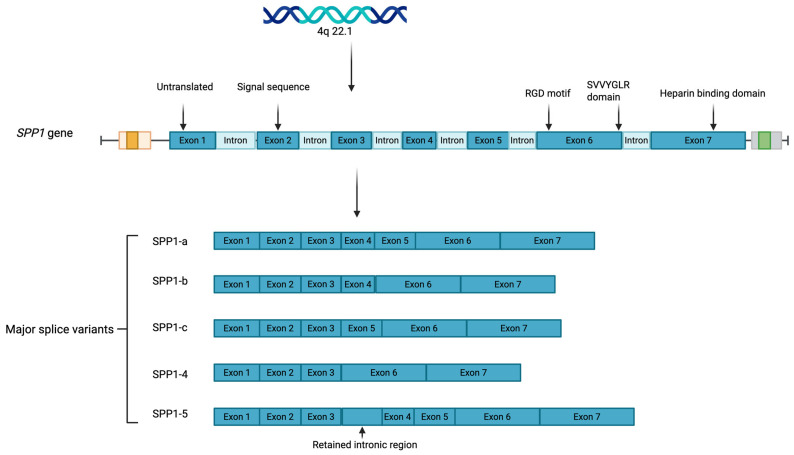
Domain structure and isoforms of SPP1. Figure created using BioRender.

Secreted phosphoprotein 1 (SPP1), also known as osteopontin (OPN) or early T lymphocyte activation protein 1 (ETA-1), is a multifunctional extracellular matrix (ECM) protein that is implicated in various physiological and pathological processes [1]. The SPP1 gene is located on human chromosome 4q22.1 and is a 314-amino-acid protein consisting of seven exons, with exon 1 being untranslated. Initially identified for its involvement in bone homeostasis, recent studies have revealed that SPP1 is also a critical mediator of stress responses, immune modulation, and tissue remodeling. Despite SPP1’s involvement in numerous pulmonary pathologies, the precise mechanistic roles of SPP1 remain incompletely understood. This complexity partly stems from the presence of distinct SPP1 isoforms (Figure 1), which harbor unique functions depending on the cell type or disease context. Alternative splicing of SPP1 generates at least five major SPP1 isoforms:

- SPP1-a, the full-length transcript containing all seven exons;
- SPP1-b, lacking exon 5;
- SPP1-c, lacking exon 4;
- SPP1-4, which lacks both exons 4 and 5;
- SPP1-5, which includes a retained intronic region between exons 3 and 4 that adds a novel exon and alters the translation start site. Subvariants of SPP1-5 (e.g., SPP1-5b through SPP1-5e) contain specific insertions or deletions in the novel exon. These splice variants differ in motifs for phosphorylation and transglutaminase crosslinking, contributing to isoform-specific functions in disease. Moreover, SPP1 exists in both intracellular and secreted forms and undergoes extensive post-translational modifications, including serine/threonine phosphorylation and glycosylation [2], which differentially influence its function. Exon 2 encodes the signal peptide necessary for secretion, while exons 3 through 7 contribute to its phosphorylation sites, calcium-binding domains, and receptor-binding motifs. The extracellular form of SPP1 primarily exerts its effects through interactions with integrins and CD44, regulating cellular functions such as migration, adhesion, and proliferation [3,4,5]. SPP1 contains a conserved Arg-Gly-Asp (RGD) motif, which facilitates integrin binding and is regulated by active thrombin cleavage [6]. The integrin-binding Arg-Gly-Asp (RGD) motif, located in exon 6, is essential for binding to αvβ3, αvβ5, and α8β1 integrins, and the SVVYGLR domain enables interaction with α4β1, α9β1, and α4β7 integrins. In addition to thrombin, matrix metalloproteinases (MMPs)—specifically MMP-3 and MMP-7—can also cleave SPP1 [7]. While most research has focused on the extracellular form, intracellular SPP1, located in the cytoplasm and nucleus, can act independently of receptor-mediated pathways, influencing processes such as calcium signaling, cytoskeletal organization, and apoptosis [8]. This dual localization raises important questions about how each form of SPP1 contributes to disease progression, either through extracellular signaling or intracellular regulation. The context of SPP1 expression—whether as part of a physiological adaptation or a pathological response—is also critical. In some instances, SPP1 upregulation may reflect an adaptive response to injury or inflammation, promoting tissue repair and cellular survival. Indeed, SPP1 has been shown to be a critical factor for lung development in mice [9]. However, SPP1 may contribute to disease processes such as fibrosis, immune dysregulation, or cancer metastasis. This multifaceted nature—being both protective and pathogenic—underscores the need for a nuanced understanding of SPP1’s isoform- and cell-specific functions, subcellular localization, and disease context.

This review will focus on the role of SPP1 in the pathogenesis of various acute and chronic lung diseases, including interstitial lung diseases (ILDs), granulomatous lung diseases, lung malignancies, airway diseases, pulmonary hypertension, and COVID-19 [10,11,12,13,14]. By exploring the diverse roles of SPP1 in these conditions, this review will highlight the potential of SPP1 as both a diagnostic and prognostic biomarker, as well as a therapeutic target in respiratory diseases, particularly for ILDs.

## 2. SPP1 in Interstitial Lung Diseases

***(i) SPP1 in idiopathic pulmonary fibrosis (IPF):*** ILD is an umbrella term for a group of disorders characterized by irreversible parenchymal fibrosis. IPF accounts for approximately 25% of ILD cases. IPF is a progressive disease with high mortality and an average life expectancy of less than three years following diagnosis [15]. Additionally, the incidence and prevalence of IPF and other ILDs continue to rise [16]. The current understanding of IPF pathogenesis suggests it begins with aberrant epithelial injury and dysregulated repair, which activates profibrotic monocyte-derived macrophages (MDMs) and (myo)fibroblasts [17]. Two approved anti-fibrotic therapies, pirfenidone and nintedanib, slow disease progression; however, neither halts nor reverses lung function decline. Therefore, there is an urgent need to identify new biomarkers and develop effective therapeutics for accurate diagnosis and disease treatment in IPF.

Plasma SPP1 levels—either alone or in combination with surfactant protein-D (SP-D) and MMP-7—have been shown to differentiate IPF from other idiopathic interstitial pneumonias (IIPs) [10]. An SPP1 concentration >6 ng/mL distinguishes IPF from other IIPs (adjusted odds ratio: 3.8, 95% confidence interval: 1.0–14.3), with 91% specificity but a relatively low sensitivity of 35%. A combined measurement of SPP1, SP-D, and MMP-7 enhances diagnostic accuracy, regardless of high-resolution computed tomography (HRCT) pattern, lung function, or smoking status. Notably, these biomarkers do not distinguish IPF from rheumatoid arthritis-associated ILD (RA-ILD), likely due to the shared usual interstitial pneumonia (UIP) pattern seen on HRCT in both conditions. Serum proteomic screening has shown that SPP1 modestly predicts three-year transplant risk (hazard ratio: 1.37, 95% confidence interval: 1.1–1.59), although it is outperformed by other markers, such as latent-transforming growth factor β-binding protein 2 [18]. Additionally, SPP1 is significantly elevated in bronchoalveolar lavage (BAL) fluid from IPF patients compared to controls [19,20]. In summary, SPP1 is elevated in both serum and BAL fluid in IPF subjects and may serve as a diagnostic or prognostic biomarker, either alone or in combination with other biomarkers.

SPP1 is upregulated in the lung tissue of bleomycin-injured wild-type mice by day 14 post-injury [21,22]. However, SPP1 expression remains unchanged in *β6*^−/−^ mice, which exhibit inflammation but are protected from fibrosis, supporting a unique role for SPP1 in tissue remodeling. While immunohistochemistry reveals SPP1 expression primarily in the bronchial epithelial cells of healthy lungs [23], recent studies have also identified SPP1+ mononuclear cells in BAL fluid from healthy individuals. Transcriptomically, these cells more closely resemble monocytes than resident alveolar macrophages and express high levels of cell–matrix interaction genes [24]. In IPF patients and bleomycin-injured mice, SPP1 is elevated in alveolar epithelial cells, macrophages, and fibroblasts—the three key cell types implicated in IPF pathogenesis [19,22,25,26,27]. Single-cell RNA sequencing (scRNA-seq) studies have repeatedly identified an abundant macrophage subset enriched in SPP1 in IPF lungs, particularly in the lower lobes. These *SPP1^high^* macrophages co-express *MERTK* and exhibit low *FABP4* levels, indicating that they originate from circulating monocytes. These cells express genes involved in stress responses and promote differentiation of fibroblasts into myofibroblasts, as demonstrated by causal modeling. Additionally, *SPP1^-/-^* mice have reduced TGF-β and MMP-2 [28] compared to wildtype mice, suggesting attenuated profibrotic responses. Smoking is a major risk factor for IPF, and studies have shown that patients with smoking-related lung diseases such as pulmonary Langerhans cell histiocytosis (PLCH) and desquamative interstitial pneumonitis (DIP) have much higher levels of SPP1 expression in BAL cells compared to those from IPF subjects [29].

Several mechanisms have been proposed to elucidate the role of SPP1 in fibrotic progression. In the fibrotic lung microenvironment, injured alveolar type II (ATII) cells secrete sonic hedgehog (SHH), which stimulates macrophage SPP1 production. SHH silencing or neutralizing antibodies of SHH reduce SPP1 production in ATII/macrophage co-culture systems [30]. SHH upregulates SPP1 via the GLI family of transcription factors, and GLI inhibition blocks this process. In vivo, SHH silencing reduces SPP1 expression and protects mice from developing fibrosis. Additionally, recombinant SPP1 can promote integrin-mediated migration, adhesion, and proliferation of fibroblasts. It also enhances epithelial cell proliferation and migration, effects that can be blocked by inhibiting SPP1-integrin and SPP1-CD44 interactions [19,22]. SPP1 modulates ECM-related proteins such as MMP-1, MMP-7, and TIMP-1 in epithelial cells. In fibroblasts, TGF-β increases SPP1 expression and drives differentiation via PI3K/AKT/mTOR. Silencing SPP1 reduces α-SMA and COL1A1 expression, and PI3K inhibition with LY294002 also lowers SPP1 levels [27]. Furthermore, silencing SPP1 blocks TGF-β-induced epithelial–mesenchymal transition (EMT) in vitro and, in bleomycin-injured mice, an important process in fibrosis progression [31]. Moreover, SPP1 also regulates T cell responses, as its original name, early T lymphocyte activation protein 1, suggests. In *Spp1*^−/−^ mice, bleomycin injury results in attenuated Th17 and IL-17-producing γδ T cell responses, correlating with less inflammation and fibrosis [32]. In summary, SPP1 is constitutively expressed in epithelial cells and macrophages and can be induced in fibroblasts and immune cells, collectively contributing to IPF pathogenesis [33].

***(ii) SPP1 in Connective Tissue Disease-related ILD (CTD-ILD):*** CTDs are systemic autoimmune conditions that frequently affect the lungs. CTD-ILDs account for approximately one-fifth of all ILDs, with ILD being a leading cause of morbidity and mortality among CTD patients [34]. SPP1 is upregulated in the serum and lungs of patients with systemic sclerosis (SSc), rheumatoid arthritis (RA), and dermatomyositis [35,36,37]. Serum SPP1 levels are elevated in both diffuse and limited forms of SSc, and higher levels are predictive of future disease progression and lung function decline in patients with SSc-ILD [35,38]. ScRNA-seq has revealed elevated *SPP1* expression in lung monocytes/macrophages from SSc-ILD subjects [35,39,40]. Immune complexes, but not lipopolysaccharide (LPS), stimulate SPP1 production in primary human monocytic cells, which is mediated by M-CSF and further augmented by IL-6 [35]. Monocyte-derived SPP1 promotes fibroblast migration, but it does not influence fibroblast proliferation or collagen production. Importantly, tocilizumab (a monoclonal IL-6 receptor antibody) has been approved for treating SSc-ILD and a study has found that serum SPP1 levels decrease following tocilizumab treatment in SSc-ILD patients. Using a single-cell assay for transposase-accessible chromatin using sequencing (ATAC-seq), several transcription factors—including MITF, TFEB, ATF6, SREBF1, BHLHE40, KLF6, ETV5, and members of the AP-1 family—have been shown to regulate SPP1-driven macrophage differentiation [39]. Interestingly, while SPP1-positive macrophages are enriched in both IPF and SSc-ILD lungs, they exhibit distinct characteristics [40]. For instance, interferon-γ (type II) signaling is upregulated in SPP1^hi^ macrophages in IPF, whereas SPP1^hi^ macrophages in SSc-ILD exhibit higher type I interferon activation. Moreover, both SPP1^hi^ and FABP4^hi^ macrophages contribute to the proliferating macrophage population in IPF, with SPP1^hi^ macrophages being the predominant contributor, whereas only FABP4^hi^ macrophages contribute to the proliferating population in SSc-ILD. In dermatomyositis-associated ILD (DM-ILD), serum SPP1 levels correlate with disease severity, especially in patients with anti-MDA5 antibodies, who are at higher risk of developing rapidly progressive ILD [37]. SPP1 levels are also increased in the serum of several other CTDs, such as Sjögren’s syndrome and systemic lupus erythematosus, although the implications of lung involvement have not been explored [41,42]. Overall, SPP1 is upregulated across multiple CTDs, particularly in cases involving fibrosing ILD, where its levels are elevated in both blood and lung tissue and correlate with disease severity. These findings suggest that SPP1 may serve as a biomarker and potential therapeutic target in CTD-ILD.

***(iii) SPP1 in Pneumoconiosis:*** Pneumoconiosis encompasses a group of ILDs caused by occupational inhalation of inorganic dusts, such as coal, silica, and asbestos. SPP1 is implicated in the pathogenesis of both silicosis and asbestosis, contributing to chronic inflammation and fibrotic remodeling. In an animal model of asbestosis, SPP1 expression is elevated in bronchiolar epithelial cells, and *Spp1*^−/−^ mice exhibit reduced inflammation and collagen gene expression [43]. Serum SPP1 levels are elevated in individuals with a history of asbestos exposure who have both fibrosis and pleural plaques, but not in those with plaques or fibrosis alone [44]. SPP1 expression is also elevated in the lungs of patients with silicosis [45]. Silica exposure increases SPP1 levels in lung tissue, BAL fluid, and serum in mice. *Spp1*^−/−^ mice are protected from developing silica-induced pulmonary fibrosis [46]. Notably, male mice have higher SPP1 levels than females, and ovariectomized female mice exhibit increased SPP1 expression and collagen deposition in the lungs compared to sham-treated female mice. Conversely, estrogen-treated male mice show reduced SPP1 expression. These findings are particularly important, as fibrotic ILDs are more prevalent and more severe in males. Studies have identified nuclear receptors within the SPP1 promoter region, and SPP1 can be induced by β-estradiol or progesterone in vivo, although this occurs in a highly isoform- and cell-context-dependent manner [47,48,49]. One mechanism by which SPP1 contributes to silicosis involves the release of macrophage-derived exosomes. A proteomic analysis of silica-exposed macrophages revealed that their exosomes contain SPP1, which promotes fibroblast-to-myofibroblast differentiation [50].

There has been a significant increase in the usage of industrial nanotubes in recent decades. In a model of multi-walled carbon nanotube (MWCNT) exposure, SPP1 enhances lung fibrosis by upregulating TGF-β and stimulating myofibroblast differentiation in vivo. Similar findings were observed in studies involving single-walled carbon nanotubes (SWCNTs), where SPP1 acts upstream of TGF-β to drive fibroblast activation and ECM deposition [51,52]. Both studies demonstrated that *Spp1*^−/−^ mice were protected from fibrosis and granuloma formation. Interestingly, SPP1-neutralizing antibodies attenuated nanotube-induced TGF-β production in macrophages, but not in epithelial cells.

***(iv) SPP1 in Noninfectious Granulomatous Lung Diseases:*** Sarcoidosis is the most common noninfectious granulomatous lung disease, characterized by immune cell-driven inflammation and granuloma formation. Elevated serum SPP1 levels have been observed in patients with sarcoidosis [53,54], and increased SPP1 expression has been detected within sarcoid granulomas, although the serum level of SPP1 may be lower than that of interstitial pneumonia (IP) patients [55]. Moreover, SPP1 elevation is more pronounced in patients with pulmonary involvement compared to those with only cutaneous manifestations. Studies investigating SPP1 gene polymorphisms and sarcoidosis susceptibility have identified several single-nucleotide polymorphisms (SNPs) associated with the disease, including rs11730582, rs11728697, and rs4754 [53,56]. As previously noted, SPP1 undergoes post-translational modifications, and research suggests that carboxyl-terminal SPP1 (C-SPP1) counteracts full-length SPP1 (F-SPP1) in sarcoidosis. While F-SPP1 promotes granuloma formation, C-SPP1 appears to inhibit this process [54]. In addition to sarcoidosis, SPP1 is also elevated in the histiocytes of various other granulomatous lung disorders [11], underscoring its broader role in granuloma formation and ILD pathogenesis. Chronic hypersensitivity pneumonitis is another ILD characterized by granuloma formation. In patients with fibrotic hypersensitivity pneumonitis, S100A^hi^ classical monocytes are present in peripheral blood, and trajectory analyses have shown that these cells differentiate into SPP1^hi^ lung macrophages [57].

Collectively, these findings highlight SPP1 as a shared effector in the pathogenesis of fibrotic ILD and a promising target for therapeutic intervention. SPP1 is present in all critical cell types that promote fibrosis, including epithelial cells, fibroblasts, and macrophages. Elevated serum and BAL fluid SPP1 levels can be a potential biomarker for ILDs, and the global knockout of *SPP1* can protect animals from developing pulmonary fibrosis in various experimental models of pulmonary fibrosis. However, the cellular origins of SPP1 remain unclear, and a cell-type-specific conditional knockout model would help advance our understanding of SPP1 in ILDs.

## 3. SPP1 in Infectious Granulomatous Lung Diseases

SPP1 expression is significantly upregulated in response to *Mycobacterium tuberculosis* infection in primary human alveolar macrophages [45]. It has been detected in macrophages, lymphocytes, and the extracellular matrix of lung tissues from patients with tuberculosis (TB). Elevated serum and plasma levels of SPP1 have been consistently reported in individuals with both active and latent TB, with levels decreasing following successful treatment for pulmonary TB [58,59,60,61,62]. Interestingly, patients co-infected with HIV and TB exhibit lower circulating SPP1 levels, possibly reflecting impaired immune activation [63]. SPP1 levels appear to correlate with disease severity. Patients who are acid-fast bacillus (AFB)-positive and those with more extensive radiographic abnormalities exhibit particularly high SPP1 levels [61,64]. Mechanistically, SPP1 may contribute to the host immune response against *M. tuberculosis* by promoting Th1 polarization through IL–12-dependent pathways [61]. Interestingly, this study also reports that serum SPP1 levels were higher in subjects with TB compared to subjects with sarcoidosis. In a case series of patients with severe Bacillus Calmette–Guérin (BCG) or nontuberculous mycobacterial (NTM) infections and underlying immune deficiencies, SPP1 expression was reduced in the lymph nodes, particularly in individuals with complete interferon-γ receptor 1 (IFNGR1) deficiency. In this cohort, higher tissue SPP1 expression was associated with better clinical outcomes, including improved survival and less severe infections, suggesting a protective role for SPP1 in T cell cytokine production, IFN- γ signaling, and macrophage recruitment during granuloma formation [45]. However, animal studies have yielded conflicting results. While some reports suggest that SPP1-deficient mice exhibit impaired immune control of mycobacterial infections, others have found no significant difference in host defense, implying that the role of SPP1 may be context-dependent and influenced by host genetics, immune status, and pathogen virulence [65,66]. Overall, SPP1 appears to modulate multiple aspects of the immune response during mycobacterial infection, particularly in granuloma formation and Th1 cytokine signaling, although its precise role in host protection remains to be fully elucidated. In other infectious lung diseases, *Spp1*^−/−^ mice have severely impaired type-1 immunity to viral and *Listeria monocytogenes* infection and do not develop sarcoid-type granulomas. This is due to reduced interleukin-12 (IL-12) and interferon γ production in macrophages, while IL-10 production is increased [67].

## 4. SPP1 in Lung and Pleural Malignancies

Elevated levels of serum or tissue SPP1 are consistently observed in various cancers, including lung, breast, prostate, and colorectal cancers. Moreover, high SPP1 expression strongly correlates with poor clinical outcomes, suggesting its role as a marker of aggressive tumor behavior [68]. SPP1 contributes to tumorigenesis and cancer progression through multiple mechanisms, including immune modulation, promotion of cell survival, proliferation, and metastasis. Its interactions with integrins are essential for cytoskeletal rearrangements that drive cell motility and activate key signaling pathways such as PI3K/AKT, nuclear factor kappa-light-chain-enhancer of activated B cells (NF-κB), and mitogen-activated protein kinase (MAPK). These pathways collectively support tumor cell proliferation, survival, and resistance to apoptosis [69,70]. For example, SPP1 activates the PI3K/AKT pathway, leading to the phosphorylation and inactivation of pro-apoptotic proteins like BCL2-associated agonist of cell death (BAD) and caspase-9, thereby enhancing tumor cell survival. Metastasis—the leading cause of cancer mortality and a common feature of lung cancer—is notably facilitated by SPP1. In non-small cell lung cancer, SPP1 is higher in the serum of patients with advanced stages (IV) cancer than in patients with stages I–III [71]. Additionally, SPP1 expression correlates with the extent of metastasis and has been proposed as a prognostic marker [72]. One mechanism by which SPP1 promotes lung cancer metastasis involves COL11A1-mediated EMT [73]. Additionally, the interaction of SPP1 with CD44 plays a pivotal role in creating a pro-metastatic tumor microenvironment. CD44 is essential for cell adhesion, migration, and invasion. The SPP1–CD44 interaction activates Rho family GTPases, which are critical for cytoskeletal reorganization and cell motility [5,74]. SPP1 also promotes angiogenesis by enhancing the blood supply to tumors via the induction of vascular endothelial growth factor (VEGF) [75]. It directly stimulates angiogenesis through the PI3K/AKT- and ERK-mediated pathways, with VEGF acting as a positive feedback signal [76]. Furthermore, SPP1 increases the expression of MMPs, particularly MMP-2 and MMP-9, which degrade the ECM and create a favorable environment for cancer cell invasion and metastasis [77,78]. Octamer-binding transcription factor 4A (OCT4A) and SPP1 are strongly co-expressed in early-stage lung adenocarcinomas and harbor a higher risk of metastasis. Silencing OCT4A reduces SPP1 expression and attenuates cancer cell migration [79]. SPP1 not only contributes to primary lung malignancy but also promotes lung metastasis of other cancers, such as melanoma [80].

Serum SPP1 levels are also significantly higher in individuals with pleural mesothelioma—a malignancy closely linked to asbestos exposure—than in those exposed to asbestos but without lung disease. In individuals with a history of asbestos exposure, a serum SPP1 level above 48.3 ng/mL yields a sensitivity of 77.6% and a specificity of 85.5% for diagnosing mesothelioma, with even higher accuracy observed in patients with stage I disease. Moreover, SPP1 levels are also elevated in the pleural fluid from mesothelioma patients compared to those with non-mesothelioma etiologies [44,81]. However, the sensitivity of serum SPP1 is lower than other biomarkers, such as serum mesothelin [82,83].

In summary, SPP1 holds promise as a diagnostic and prognostic biomarker for lung and pleural cancers. Its elevated expression promotes tumor progression through diverse mechanisms, including angiogenesis, EMT, MMP activation, and modulation of key signaling pathways essential for cell survival and proliferation, making it a compelling target for future cancer therapies.

## 5. SPP1 in Obstructive Lung Diseases and Reactive Airway Diseases

SPP1 is elevated in the distal airways of subjects with chronic obstructive pulmonary disease (COPD), including increased expression in goblet and club cells. Elevated levels of SPP1 in sputum are associated with neutrophilic inflammation and the extent of radiographic emphysema, the most common subtype of COPD, highlighting its correlation with disease severity [84]. Furthermore, serum SPP1 levels are increased in COPD patients with frequent exacerbations and during acute exacerbations, suggesting its potential as a biomarker for monitoring disease activity and exacerbation risk [85,86]. Smoking, the primary risk factor for COPD, can upregulate SPP1 expression in airway epithelial cells in vitro [87] and in BAL cells in vivo [29]. SPP1 expression is elevated in various cell types in COPD. For example, lung dendritic cells from patients with emphysema exhibit higher SPP1 expression compared to controls. This finding was further validated in a cigarette smoke model, where SPP1 levels increased in lung antigen-presenting cells (APCs) after cigarette smoke exposure in wild-type mice. Additionally, *Spp1*^−/−^ mice were protected from developing emphysema after smoke exposure [14]. Mechanistically, SPP1 is required for inducing Th1 and Th17 differentiation from naïve T cells by downregulating the expression of the transcription factor IFN regulatory factor 7 (IRF7) in lung APCs. IRF7 acts as a negative regulator of IL-1β and IL-6 production, and SPP1 inhibits IRF7, thereby promoting the production of these pro-inflammatory cytokines. *Ada*^−/−^ mice is another model of COPD, and *Spp1*^−/−^*Ada*^−/−^ double knockout mouse leads to reduced histopathologic and biochemical features of pulmonary inflammation and emphysema, primarily by attenuating neutrophil recruitment and SPP1-dependent MMP9 production [88]. Interestingly, SPP1 does not affect MMP-12 production, another MMP critical for COPD pathogenesis, and *Spp1*^−/−^*Ada*^−/−^ double knockout mice displayed no changes in myofibroblast activity. Moreover, SPP1 has been implicated in the upregulation of haptoglobin expression via the PI3K/AKT pathway, with PI3K inhibition reducing SPP1-induced haptoglobin production, positioning it as a potential therapeutic target for acute exacerbations of COPD [89].

In asthma, SPP1 levels are elevated in the serum, sputum, and bronchial tissue of patients, with studies linking these levels to disease severity and the timing of onset [90,91,92,93,94,95,96]. While BAL fluid and serum SPP1 levels are significantly increased in all asthmatic patients in the steady state, serum SPP1 levels were unexpectedly reduced during exacerbation [92]. Additionally, serum SPP1 levels are significantly higher in subjects with late-onset asthma compared to those with early-onset asthma. These highlight the potential of SPP1 as a biomarker for diagnosis, monitoring, and phenotyping. SPP1 is expressed in alveolar epithelial cells, macrophages, airway cells, vascular smooth muscle cells, fibroblasts, T cells, and mast cells in asthmatic subjects [92,95,97]. In allergic responses, SPP1 may have opposing effects, with a proinflammatory response at primary systemic sensitization and an anti-inflammatory response during secondary pulmonary challenge. This is mainly regulated by T(H)2-suppressing plasmacytoid dendritic cells (DCs) during primary sensitization and T(H)2-promoting conventional DCs during secondary antigenic challenge. In an inhaled ovalbumin (OVA) model of chronic allergen-induced asthma, SPP1 expression correlates with collagen content and peribronchial smooth muscle area [90,95]. In the same model, the genetic knockout of *Spp1* results in reduced airway remodeling and lower levels of Th2 cytokines, including IL-4 and IL-13, in both lung tissue homogenates and BAL fluid, suggesting that SPP1 plays a key role in the inflammatory response and structural remodeling associated with asthma [90]. There is conflicting data regarding SPP1 overexpression in allergic airway disease. Recombinant SPP1 (rSPP1) administration increases IL-13 and MMP-9 in the lungs of *SPP1*^−/−^ mice and exacerbates airway hyperresponsiveness [95]. However, another study showed that administering rSPP1 during pulmonary secondary antigenic challenge decreased established T(H)2 responses and protected mice from developing allergic responses. One of the proposed mechanisms is that rSPP1 induces proliferation of human bronchial smooth muscle by binding to αvβ3 integrin receptor and activating the PI3K/AKT pathway. Interestingly, SPP1^+^-recruited macrophages are also present in allergic asthmatic patients, suggesting a potential conserved mechanism of lung injury and repair across a spectrum of lung diseases [97].

## 6. SPP1 in COVID-19-Associated Lung Disease

Coronavirus disease 2019 (COVID-19), caused by the SARS-CoV-2 virus, can lead to acute lung injury and, in some cases, long-term pulmonary complications including fibrosis. SPP1 is known to be highly expressed in alveolar macrophages from patients with acute respiratory distress syndrome (ARDS) and mice that received intratracheal instillation of LPS [98]. ScRNA-seq analyses of BAL and autopsy lung tissue from patients with severe COVID-19 have revealed a marked increase in SPP1 expression, particularly in macrophages [12,36,99,100]. Spatial transcriptomic analyses also confirmed the presence of SPP1^hi^ macrophages in subjects with severe COVID-19 and a murine model of COVID-19 infection [99,101]. These SPP1^hi^ macrophages, which share transcriptional features with those found in IPF and other fibrotic ILDs, suggest a shared fibrotic signature across distinct etiologies. Indeed, BAL fluid SPP1 levels are predictive of post-COVID-19 ILDs [102], and profibrotic monocyte-derived alveolar macrophages, which are SPP1^hi,^ are expanded in patients suffering from post-acute sequelae of COVID-19 (PASC) with persistent respiratory symptoms [103].

## 7. Future Research

Despite recent advances in research, several critical knowledge gaps remain. The precise cellular sources of SPP1 are not fully defined, and global knockout models fail to capture the heterogeneity of its functions across cell types and disease states. Moreover, the isoform-specific effects of SPP1 and its post-translational modifications in acute and chronic lung diseases remain largely unexplored. Future research should prioritize (1) elucidating the cell-type-specific functions of SPP1 and performing cell type-specific studies using conditional knockout or lineage-tracing models to unravel the roles of SPP1 in epithelial, immune, and mesenchymal compartments; (2) isoform- and context-specific investigations to delineate the functional differences between full-length and cleaved forms of SPP1 across disease subtypes; (3) dissecting the upstream regulators and downstream effectors of SPP1 in fibrotic and inflammatory niches; (4) investigating the interplay between SPP1 and other fibrotic mediators in multi-omic frameworks to better define molecular endotypes; (5) longitudinal clinical studies to validate SPP1 as a prognostic biomarker and to define thresholds for clinical utility in diagnosis, risk stratification, and therapeutic monitoring; and (6) exploring therapeutic modulation of the SPP1 axis, including the development of small molecule inhibitors, neutralizing antibodies, or gene-silencing approaches, particularly in early-stage disease or in combination with existing antifibrotic agents.

## 8. Conclusions

In conclusion, SPP1 represents a unifying effector across diverse forms of lung diseases, particularly ILD (see Table 1). Its pleiotropic roles in modulating inflammation, ECM deposition, immune cell recruitment, and tissue remodeling underscore its potential as both a biomarker and a therapeutic target. A deeper understanding of SPP1 biology may pave the way for personalized interventions and improved outcomes for patients suffering from these debilitating lung diseases.

## Figures and Tables

**Table 1:** The role of SPP1 in various lung diseases.

Lung Disease			SPP1 Function/Role
**Interstitial Lung Disease**	Idiopathic Pulmonary Fibrosis		Drives fibrosis via SHH-induced macrophage activation, TGF-b signaling, and integrin-mediated fibroblast proliferation. Promotes EMT and modulates immune cell responses.
	CTD-associated ILD		Stimulates fibroblast migration via IL-6 and M-CSF-activated macrophages. Regulated by immune complexes. SPP1 levels decrease with IL-6 pathway inhibition (tocilizumab).
	Pneumoconiosis		Mediates TGF-b signaling, macrophage-derived exosomes, and myofibroblast differentiation.
	Granulomatous Lung Diseases (noninfectious)		Facilitates granuloma formation through full-length isoform; antagonized by C-terminal fragment. Upregulated in macrophages within granulomas.
Infectious Granulomatous Diseases (e.g., TB)			Enhances Th1 immune response via IL-12 pathways. Expression correlates with disease severity. Modulates macrophage recruitment and IFN-g signaling.
Lung and Pleural Malignancies			Activates PI3K/AKT, NF-kB, and MAPK pathways. Promotes EMT, angiogenesis, and metastasis via integrin/CD44 interaction. Upregulates VEGF and MMPs.
Obstructive Lung Disease and Reactive Airway Disease		COPD	Drives Th1/Th17 inflammation by inhibiting IRF7 in lung APCs. Increases IL-1b, IL-6, and MMP-9. Induced by cigarette smoke exposure.
		Asthma	Mediates airway remodeling and Th2 inflammation. Activates PI3K/AKT in bronchial smooth muscle and influences IL-4, IL-13 expression.
COVID-19-associated Lung Disease			SPP1^hi^ macrophages in severe and post-COVID lungs drive profibrotic programs; share features with IPF macrophages.

## Data Availability

No new data were created or analyzed in this study.
